# The role of procalcitonin in the diagnosis of bacterial infection after major abdominal surgery: Advantage from daily measurement

**DOI:** 10.1097/MD.0000000000009496

**Published:** 2018-01-19

**Authors:** Silvia Spoto, Emanuele Valeriani, Damiano Caputo, Eleonora Cella, Marta Fogolari, Elena Pesce, Maria Tea Mulè, Mariacristina Cartillone, Sebastiano Costantino, Giordano Dicuonzo, Roberto Coppola, Massimo Ciccozzi, Silvia Angeletti

**Affiliations:** a Internal Medicine Department, University Campus Bio-Medico of Rome, Rome; b Internal Medicine Department, University G. D’Annunzio, Chieti; c Department of Surgery, University Campus Bio-Medico of Rome; d Department of Public Health and Infectious Diseases, Sapienza University of Rome; e Unit of Clinical Laboratory Science, University Campus Bio-Medico of Rome, Rome, Italy.

**Keywords:** early diagnosis, PCT, surgical infections

## Abstract

Postsurgical infections represent an important cause of morbidity after abdominal surgery. The microbiological diagnosis is not achieved in at least 30% of culture with consequent worsening of patient outcome. In this study, procalcitonin measurement, during the first 3 days after abdominal surgery, has been evaluated for the early diagnosis of postsurgical infection.

Ninety consecutive patients subjected to major abdominal surgery at the University Campus Bio-Medico of Rome, have been included. PCT concentrations were measured by time-resolved amplified cryptate emission (TRACE) assay at admission and at the first, second, and third day after surgery. PCT levels were compared using the Mann–Whitney test and by ANOVA test for variance analysis. Receiver operating characteristic (ROC) analysis was performed to define the diagnostic ability of PCT in case of postsurgical infections.

PCT values resulted significantly different between patients developing or not developing postsurgical infections. PCT >1.0 ng/mL at first or second day after surgery and >0.5 ng/mL at third day resulted diagnostic for infectious complication, whereas a value <0.5 ng/mL at the fifth day after surgery was useful for early and safety discharge of patients.

In conclusion, PCT daily measurement could represent a useful diagnostic tool improving health care in the postsurgical period following major abdominal surgery and should be recommended.

## 1 Introduction

Postsurgical infections, a major cause of morbidity after abdominal surgery, are classified into surgical site infections (SSIs) and distant infections such as, respiratory tract infections, urinary tract infections, catheter associated infections, and sepsis.^[1]^

Several risk factors such as age, sex, body mass index (BMI), comorbidities (diabetes or organ-failure), previous chemotherapy or radiotherapy, have been associated with a higher probability to develop this complication.^[2,3]^ In many studies, a relationship between traumatic or surgical insults and the development of localized or disseminated infections and multiple organ failure (MOF) has been reported.^[4]^ The prompt diagnosis of postsurgical infections is crucial for optimal patient management by early diagnosis of infectious complication, adequate antibiotic treatment administration; early oral intake resume and appropriate computed tomography (TC) scan indication and early discharge.

The microbiological diagnosis achieved by the isolation of the causative pathogen is not always possible; at least 30% of culture remains negative. The lack of detection of the causing microorganisms can worsen patient outcome increasing hospital length of stay and costs.^[5]^ Inflammatory markers such as C-reactive protein (CRP) and procalcitonin (PCT) have been previously evaluated in the diagnosis of bacterial infection.^[6,7]^ Although used for many years, CRP specificity in the diagnosis of bacterial infections versus inflammation has been often questioned.^[7–10]^ CRP is an acute phase protein promptly released during inflammation condition. Since systemic bacterial infection is often associated with inflammatory reaction, it represents an indirect marker of infection and inflammation. It is often elevated in noninfectious as well as in infectious inflammatory response without any specificity for bacterial infections.

Conversely, PCT rapidly increases within 3 to 6 hours from bacterial infection^[11]^ showing the ability to differentiate between localized and systemic infections, as previously reported.^[12]^ Huang et al in a prospective study evaluated the use of a PCT-based algorithm to guide antibiotic therapy after urgent abdominal surgery. These authors concluded that PCT reduced the duration of antibiotic treatment thus avoiding the risks of side effects and the development of multidrug resistant strains.^[13]^ In a recent meta-analysis to evaluate PCT ability to detect bacterial peritonitis, PCT resulted a sensitive and specific marker useful besides clinical findings for the diagnosis of bacterial peritonitis.^[14]^ Recently, the ability of PCT to differentiate infectious from not-infectious SIRS with more specificity than other biomarkers such as CRP and white blood cells (WBCs) count, has been reported.^[5,15–17]^

The present study was performed to evaluate the advantage from PCT daily measurement after major abdominal surgery. The principal aim was the early diagnosis of postsurgical infections to achieve adequate antimicrobial therapy administration, appropriate timing for imaging exams, and early oral intake resume.

## 2 Materials and methods

### 2.1 Patient selection and study design

The study was performed on 90 consecutive patients undergoing to major abdominal surgery at the Department of General Surgery of the University Campus Bio-Medico of Rome, during 18 months. Informed consent was obtained from all patients prior enrollment to the study. The Ethical Committee of the University Hospital Campus Bio-Medico of Rome approved the study.

Inclusion criteria were as follows: patient candidate for elective major abdominal surgery by laparotomy or laparoscopy technique for benign or neoplastic causes, absence of infection and preoperative PCT value <0.5 ng/mL. Exclusion criteria were: absence of informed consent; age <18 years; evidence of infectious disease or antibiotic therapy on course or preoperative PCT value ≥0.5 ng/mL; preoperative infections treated with steroids, presence of immunosuppressive diseases; thyroid, lung, pancreatic cancer; major trauma; severe burn; cardiac shock; emergency surgery. At inclusion, demographic characteristics were recorded, such as age, gender, prior or current use of antibiotics, steroid or other immunosuppressive medications or treatments, immune status, comorbidities, and clinical presentation. Risk assessment was performed by calculation of the ASA (American Society of Anesthesiologists) score. Clinical data, such as temperature, heart and respiratory rate, blood pressure, urinary output, and mental status were recorded. Laboratory data as WBC, absolute number of neutrophil leukocytes, CRP, and PCT levels, indicating the dynamics of inflammatory response and the possibility of infectious complication were collected.

In presence of clinical signs of infection, depending on the site, microbiological investigations were carried out by blood culture, culture of biological materials collected from abdominal abscesses; wound, urine, sputum culture, and urine antigen detection of *Legionella pneumophila* Type 1 or *Streptococcus pneumoniae*. Moreover, empiric antibiotic therapy was administrated whereas a targeted treatment was decided based on the culture positivity. In presence of abscess, a drainage was positioned.

Patients included in the study received antibiotic prophylaxis with cefazolin (1 g i.v.) or with erythromycin in case of allergy, within 1 hour before surgical incision.

### 2.2 PCT measurement at admission (presurgery) and at the first, second, and third day after major abdominal surgery

PCT plasma concentrations were measured by an automated Kryptor analyzer, using a time-resolved amplified cryptate emission (TRACE) technology assay (Kryptor PCT; Brahms AG, Hennigsdorf, Germany), with commercially available immunoluminometric assays (Brahms AG).^[15–17]^

### 2.3 Statistical analysis

Data have been analyzed using Med-Calc 11.6.1.0 statistical package (MedCalc Software, Mariakerke, Belgium). Plasma levels of PCT recorded presurgery and at the first, second, and third day after surgery in patients developing postsurgical infections and not were compared using the nonparametric Mann–Whitney test, *P*-value <.05 were considered as significant. Receiver operating characteristic (ROC) analysis was performed to define the ability of PCT to differentiate postsurgical infected and not infected patients. ANOVA test for variance analysis in case of multiple repeated measurement has been used for PCT values at different time points (preoperative; at first, second, and third postsurgery day) comparison.

## 3 Results

### 3.1 Patients characteristics

The demographic and clinical characteristics of the 90 patients included in the study are reported in Table 1. Age, sex, smoking, BMI >30 or <18 and the presence of comorbidities were not significantly associated with the development of postsurgical infections. Patients with ASA score 3 and 4 developed postsurgical infections in 23/90 (64%) and 32/90 (16%) cases, respectively. A statistically significant difference has not been reached but a clinical trend for the postsurgical infection development has been observed (*P* = .06).

**Table 1:**
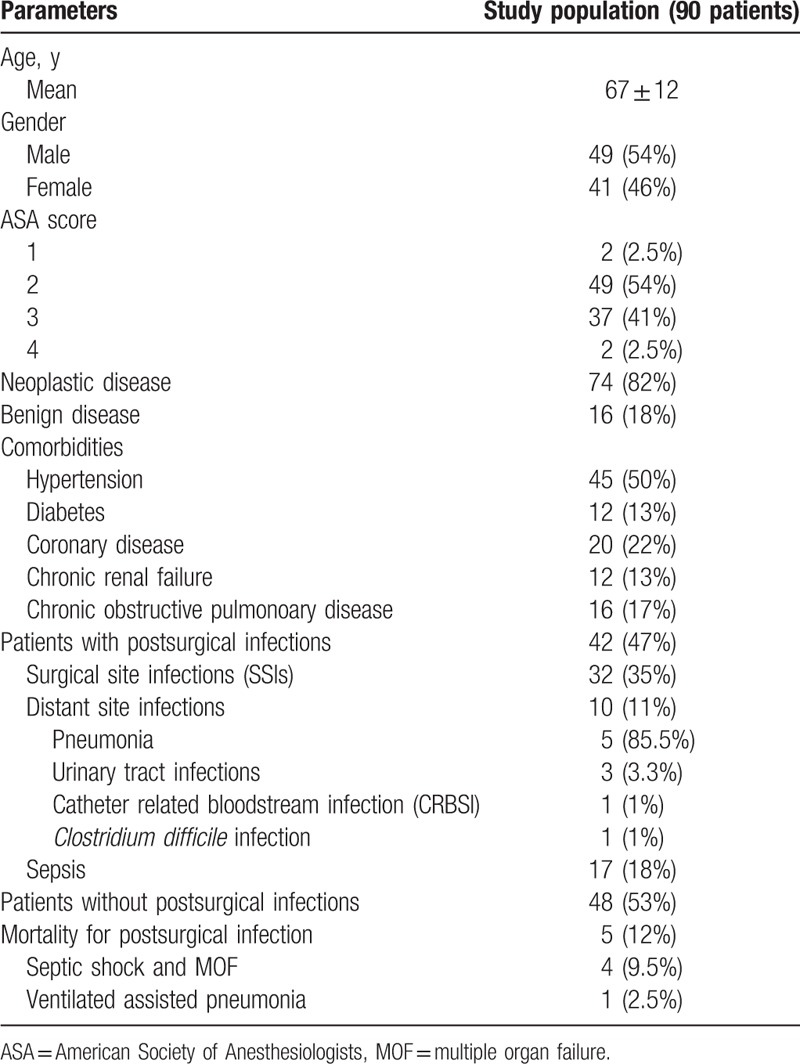
Demographic and clinical characteristics of the study population.

Patients received laparotomy in 50/90 (55.5%) and laparoscopy in 40/90 (44.5%) of cases. A positive trend for the presence of infectious complications was evidenced in case of laparotomy (*P* = .06). In 65/90 (72%) of cases colorectal surgery was performed. Postsurgery infection was developed in 42/90 (47%) of patients undergoing major abdominal surgery. In 29/42 (69%) of cases the microbiological isolation was achieved. The most represented species were *Enterococcus* spp., *Escherichia coli*, *Pseudomonas aeruginosa*, *coagulase negative staphylococci* (*CONS*), and *Klebsiella* spp. (Table 2).

**Table 2:**
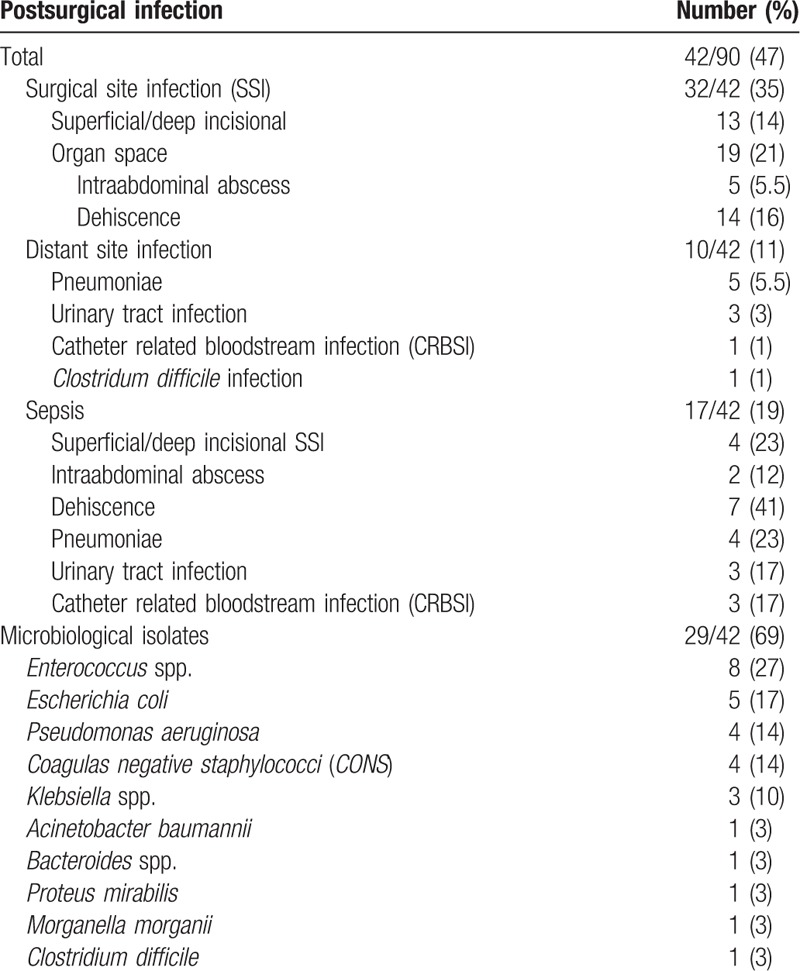
Postsurgical infections characteristics.

In 35/90 patients (32%) SSIs, in 10/90 (11%) distant site infection and in 17/90 (18%) sepsis, were diagnosed (Table 2). On average, postsurgical infection were clinically evident at the fourth day after surgery. SSIs were classified as superficial/deep incisional in 13/90 (13%) and organ/space SSIs in 19/90 (21%) of patients. Organ/space infections were treated by drainage from the abdominal collection in 5 cases (5.5%) and by surgery consequently to anastomotic dehiscence in 14 cases (16%). Postsurgical sepsis was diagnosed in 17/90 (19%) patients with intraabdominal source of infection in 9/90 (11%) (7 anastomotic dehiscence and 2 abdominal collections), with superficial/deep incisional SSIs in 4/90 (4.4%) patients (3 superficial and 1 deep SSIs) and with distant site infections in 10/90 (11%) patients (5 pneumonia, 3 urosepsis, 1 catheter related bloodstream (CRBSI) infection, and 1 *Clostridium difficile* infection) (Table 2). Five on 42 patients developing surgical infections dead (12%) (Table 1). Death was caused by septic shock and MOF consequently to anastomotic dehiscence in four cases and ventilator associated pneumonia in 1 case (Table 1).

### 3.2 PCT values measured at admission (presurgery) and at the first, second, and third day after major abdominal surgery in study population

**Figure 1:**
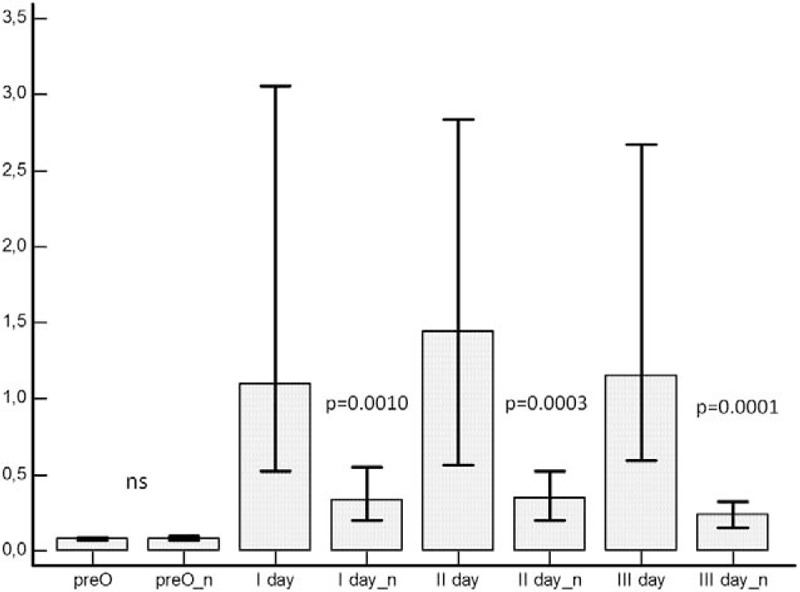
Mann–Whitney test. PCT comparison between complicated and not complicated (n) patients at different time points: preoperative (pre-O); first, second, and third day after surgery (I day, II day, and III day).

Median values, interquartile ranges (25th percentile and 75th percentile), and Mann–Whitney comparison of PCT values registered at the different time points (presurgery, first, second, and third day after surgery) in the study population are reported in Table 3 and represented as box plots in Fig. 1. Statistically significant difference in PCT values between patients developing or not developing postsurgical infections were found. Any difference was evidenced between the presurgery values in the 2 groups of patients (Table 3 and Fig. 1). Any significant difference in PCT values between surgical infections occurring after laparotomy or laparoscopy was found.

**Table 3:**
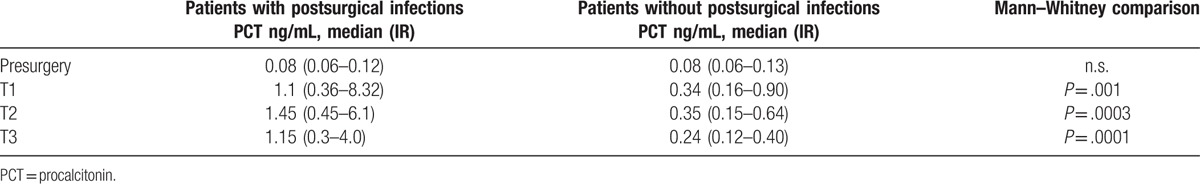
Mann–Whitney comparison: PCT values at admission (presurgery), first, second, and third day after surgery (T1, T2, and T3) in patients developing postsurgical infection and not.

### 3.3 ROC curves and areas under the curves (AUCs) analysis

**Figure 2:**
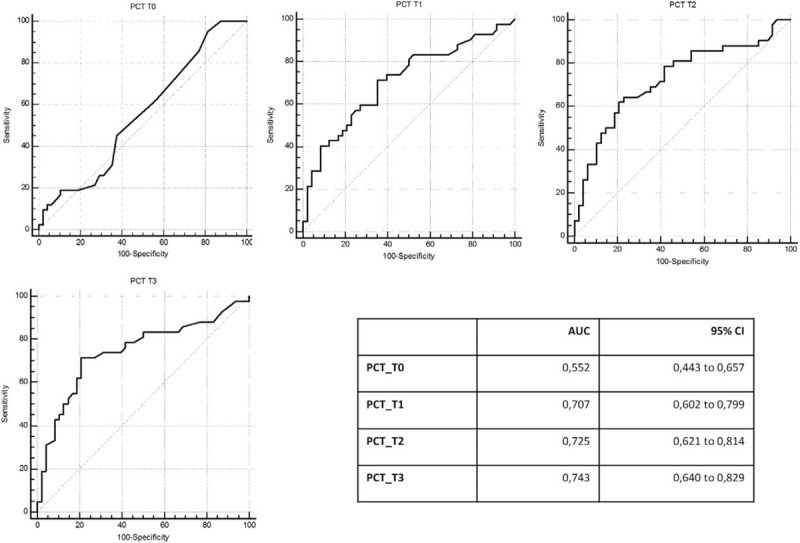
ROC curve analysis: PCT ability to differentiate infected and not infected patients at different time points preoperative day (T0), first, second, and third postoperative days (T1, T2, and T3). Area under the ROC curve (AUC) values and 95% confidence interval (CI).

**Figure 3:**
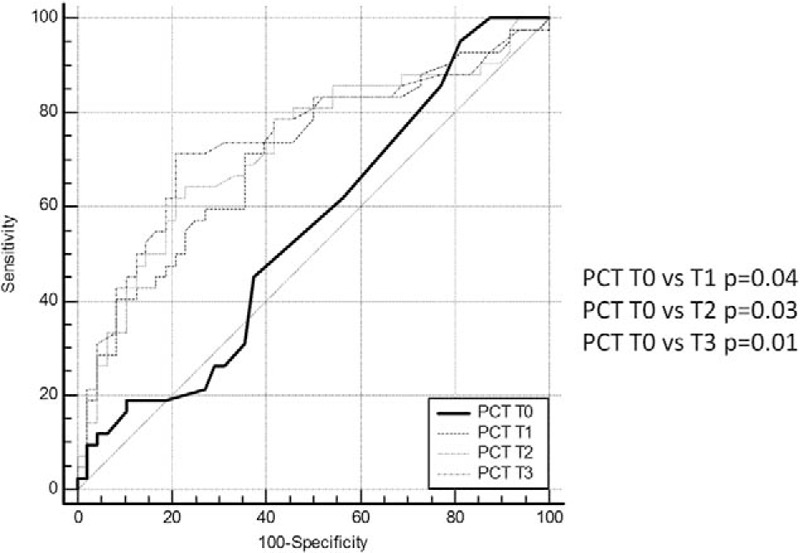
ROC curve comparison: PCT ability to differentiate infected and not infected patients at different time points preoperative day (T0), first, second, and third postoperative days (T1, T2, and T3).

ROC curve of PCT registered presurgery and at the different time points (first, second, and third day after surgery) are represented in Fig. 2. PCT is able to identify patients with postsurgical infections since the first day after surgery. AUCs analysis showed that PCT AUC values registered at the first, second, and third day after the surgical procedure are significantly different from those observed at the presurgery time point (Fig. 3). At ROC curve analysis, the diagnostic PCT cut-off value resulted >0.5 ng/mL. In Table 4, sensitivity and specificity related to three different PCT values, 0.5, 1.0, and 2.5 ng/mL are reported for each time point of measurement, first (T1), second (T2), and third day (T3) after surgery. Increased PCT values correspond to enhanced diagnostic specificity. The cut-off of 1.0 ng/mL at T1 and T2 resulted the best combination between sensitivity and specificity (T1: sensitivity 50%–specificity 80%; T2: sensitivity 57%–specificity 81%), whereas at T3 the best cut-off was 0.5 ng/mL (sensitivity 71%–specificity 79%). In a subgroup of patients, PCT was measured also at the fifth day after surgery. PCT value <0.5 ng/mL at the fifth day showed a high negative predictive value with sensitivity of 54% and specificity of 97%.

**Table 4:**
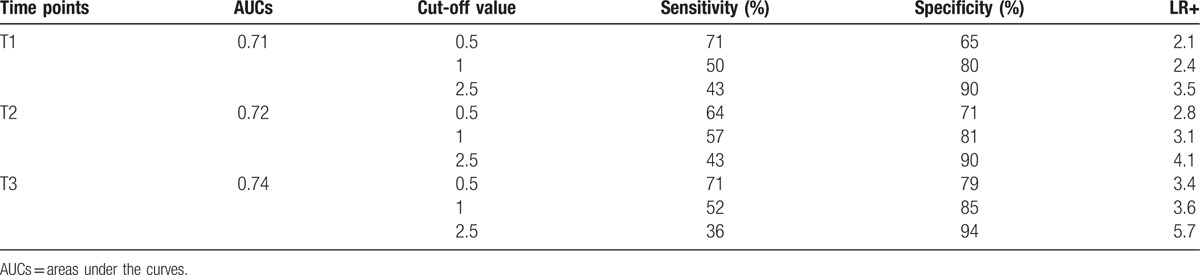
AUCs, sensitivity, specificity, and positive likelihood ratio (LR+) using the specific cut-off values for PCT, at the different time points after surgery (first, second, and third day after surgery, T1, T2, and T3, respectively) as suggested by ROC curve analysis in patients with postsurgical infections.

### 3.4 ANOVA test for variance analysis in case of multiple repeated measurement

**Figure 4:**
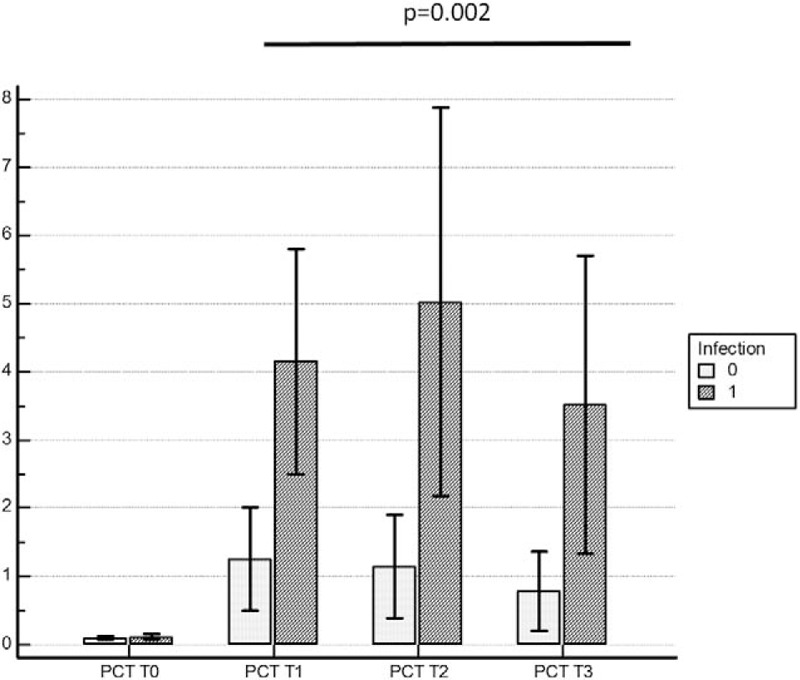
ANOVA analysis of variance on PCT measurement at different time points in the 2 groups of patients with and without infection (1,0).

Variance analysis to compare PCT values registered at different time point in patients developing postsurgical infections and patients without any evidence of postsurgical infections, showed a statistically significant difference between the 2 groups of patients at the first, second, and third day after surgery (*P* = .002). Any statistically significant difference was found presurgery between the 2 groups (Fig. 4 panel A).

ANOVA test variance performed on PCT values registered at the different time points in the group of patients with postsurgical sepsis showed a statistically significant difference in these patients from those not developing sepsis (Fig. 4 panel B).

## 4 Discussion

Postsurgical infections represent the most important cause of morbidity after major abdominal surgery.^[1]^ The surgical insult predisposes to infectious complications and MOF^[4]^ influencing the patients prognosis, hospital length of stay and cost. Patient outcome can be improved by early diagnosis and prompt treatment of postsurgical infections. In the period immediately consequent to a surgical procedure, infection diagnosis can represents a challenge because clinical signs could be vague and misleading for the clinician. For these reason diagnosis could be delayed increasing morbidity and worsening patient prognosis.

PCT has been largely investigated in sepsis and septic shock and to guide antibiotic therapy stewardship demonstrating the ability to differentiate infectious from not-infectious SIRS.^[5,15–21]^

Few studies are available in postsurgical settings and the specific cut-off values have not been clearly defined, yet.^[13,14,22–24]^

After 6 hours from an insult, the plasmatic level of PCT increases reaching a plateau between 8 and 24 hours and decreasing rapidly if the insult has disappeared. This rapid kinetic could be useful to identify infectious complication even within the first postsurgery 24 hours.^[25]^ After 24 hours from the surgery, PCT values ≥0.5 ng/mL could suggest the persistence of the insult for a possible postsurgical infections evolution.^[26]^ Meisner et al^[27]^ reported increasing values of PCT at the first and second days after major abdominal surgery caused by bacterial contamination occurring during the surgical procedure and the anastomosis preparation. This contamination could predispose to localized or systemic infection development. PCT determination immediately after surgery could indicate how much that procedure was dirty and at a risk for infectious complication.

Data from the present study showed increased PCT values in all patient during the first 24 hours after surgery, independently from infectious complication development. According with other authors, ROC curve analysis confirmed the ability of PCT to identify patients with postsurgical infections since the first day after surgery. The persistence of elevated values of PCT even in the second and third days after surgery correlated significantly with the clinical evidence of postsurgical infections at the fourth day after surgery, in agreement with other authors.^[27]^

The definition of a correct cut-off value for postsurgical infections is important to avoid overtreatment or missed diagnosis. The PCT cut-off value of 0.5 ng/mL is currently used for sepsis diagnosis in not-surgical patients, but it could be too sensitive in surgical setting where the bacterial contamination induces for itself the marker elevation. In this case, using higher PCT cut-off value to increase specificity could be appropriate. Mokart et al^[28]^ suggested a predictive cut-off for postsurgical infections at 1.1 ng/mL for PCT measured in the first day after surgery. Oberhofer et al^[29]^ reported a cut-off value of 1.34 ng/mL during the second day after surgery as diagnostic of postsurgical complications. According with these authors, data from the present study confirmed that increased PCT cut-off value enhances the diagnostic specificity of postsurgical infections. The cut-off value >1.0 ng/mL was considered diagnostic since the first day after surgery; according with other authors this cut-off showed the best combination of sensitivity and specificity at T1 as well as at T2 time points.^[28,29]^

The cut-off value of 0.5 ng/mL could be useful in the third day after surgery to predict infection complication occurring in third day. Garcia-Granero et al^[30]^ reported that a cut-off of 0.31 ng/mL in fifth day showed 100%sensitivity, 72% specificity, 100% negative predictive value, and 17% positive predictive value. According with these authors, PCT >0.5 ng/mL at the third day after surgery (T3 time point) should be diagnostic of postsurgical infections showing the best combination of sensitivity and specificity.

PCT values lower than 0.5 are very useful for early discharge of patients in fifth day after surgery because it shows a high negative predictive value in a subgroup of 53 patients where PCT was measured until the fifth day after surgical procedure.

Data from the present study showed that the early increase of PCT and its persistence during the following 72 hours after major abdominal surgery could represent a useful diagnostic tool to select patients at risk for infectious postsurgical complications. For these patients, a strict clinical and imaging follow-up should be reserved to promptly evidence the possible infection before its clinical manifestation.

PCT daily measurement in the first 3 days after major abdominal surgery, associated with clinical, microbiological, and imaging evaluation should be recommended. This algorithm should be useful for the early diagnosis of postsurgical infectious complications potentially lethal, as sepsis; the early and appropriate use of antibiotic therapy; the early and safety oral intake resume; the adequate indication to perform CT scan; the patient length of stay and health care costs decrease.

In conclusion, PCT daily measurement could represent a useful diagnostic tool improving health care patient in the postsurgical period following major abdominal surgery.

## References

[1] Owens CD Stoessel K Surgical site infections: epidemiology, microbiology and prevention. J Hosp Infect 2008;70:3–10. 19022115 10.1016/S0195-6701(08)60017-1

[2] Iwamoto K Ichiyama S Shimokata K Postoperative pneumonia in elderly patients: incidence and mortality in comparison with younger patients. Intern Med 1993;32:274–7. 8358115 10.2169/internalmedicine.32.274

[3] Dilworth JP White RJ Postoperative chest infection after upper abdominal surgery: an important problem for smokers. Respir Med 1992;86:205–10. 1620907 10.1016/s0954-6111(06)80056-9

[4] Angele MK Faist E Clinical review: immunodepression in the surgical patient and increased susceptibility to infection. Crit Care 2002;6:298–305. 12225603 10.1186/cc1514 PMC137309

[5] Wacker C Prkno A Brunkhorst FM Procalcitonin as a diagnostic marker for sepsis: a systematic review and meta-analysis. Lancet Infect Dis 2013;13:426–35. 23375419 10.1016/S1473-3099(12)70323-7

[6] Sheldon J Riches P Gooding R C-reactive protein and its cytokine mediators in intensive-care patients. Clin Chem 1993;39:147–50. 8419041

[7] Carrigan SD Scott G Tabrizian M Towards resolving the challenges of sepsis diagnosis. Clin Chem 2004;50:1301–14. 15166107 10.1373/clinchem.2004.032144

[8] Kofoed K Andersen O Kronborg G Use of plasma C-reactive protein, procalcitonin, neutrophils, macrophage migration inhibitory factor, soluble urokinase-type plasminogen activator receptor, and soluble triggering receptor expressed on myeloid cells-1 in combination to diagnose infections: a prospective study. Crit Care 2007;11:R38. 17362525 10.1186/cc5723 PMC2206456

[9] de Kruif MD Limper M Gerritsen H Additional value of procalcitonin for diagnosis of infection in patients with fever at the emergency department. Crit Care Med 2010;38:457–63. 20083920 10.1097/CCM.0b013e3181b9ec33

[10] Simon L Gauvin F Amre DK Serum procalcitonin and C-reactive protein levels as markers of bacterial infection: a systematic review and meta-analysis. Clin Infect Dis 2004;39:206–17. 15307030 10.1086/421997

[11] Uzzan B Cohen R Nicolas P Procalcitonin as a diagnostic test for sepsis in critically ill adults and after surgery or trauma: a systematic review and meta-analysis. Crit Care Med 2006;34:1996–2003. 16715031 10.1097/01.CCM.0000226413.54364.36

[12] Angeletti S Spoto S Fogolari M Diagnostic and prognostic role of procalcitonin (PCT) and MR-pro-Adrenomedullin (MR-proADM) in bacterial infections. APMIS 2015;123:740–8. 26058482 10.1111/apm.12406

[13] Huang TS Huang SS Shyu YC A procalcitonin-based algorithm to guide antibiotic therapy in secondary peritonitis following emergency surgery: a prospective study with propensity score matching analysis. PLoS ONE 2014;9:e90539. 24594916 10.1371/journal.pone.0090539 PMC3942439

[14] Yang SK Xiao L Zhang H Significance of serum procalcitonin as biomarker for detection of bacterial peritonitis: a systematic review and meta-analysis. BMC Infect Dis 2014;14:452. 25145785 10.1186/1471-2334-14-452 PMC4155125

[15] Angeletti S Battistoni F Fioravanti M Procalcitonin and mid-regional pro-adrenomedullin test combination in sepsis diagnosis. Clin Chem Lab Med 2013;51:1059–67. 23072859 10.1515/cclm-2012-0595

[16] Angeletti S Dicuonzo G Fioravanti M Procalcitonin, MR-proadrenomedullin, and cytokines measurement in sepsis diagnosis: advantages from test combination. Dis Markers 2015;2015:951532. 26635427 10.1155/2015/951532 PMC4655267

[17] Angeletti S Ciccozzi M Fogolari M Procalcitonin and MR-proAdrenomedullin combined score in the diagnosis and prognosis of systemic and localized bacterial infections. J Infect 2016;72:395–8. 26723912 10.1016/j.jinf.2015.12.006

[18] Assink-de Jong E de Lange DW van Oers JA Stop Antibiotics on guidance of Procalcitonin Study (SAPS): a randomised prospective multicenter investigator-initiated trial to analyse whether daily measurements of procalcitonin versus a standard-of-care approach can safely shorten antibiotic duration in intensive care unit patients—calculated sample size: 1816 patients. BMC Infect Dis 2013;13:178. 23590389 10.1186/1471-2334-13-178 PMC3637799

[19] Bouadma L Luyt CE Tubach F Use of procalcitonin to reduce patients’ exposure to antibiotics in intensive care units (PRORATA trial): a multicentre randomised controlled trial. Lancet 2010;375:463–74. 20097417 10.1016/S0140-6736(09)61879-1

[20] De Santis V Corona A Procalcitonin to guide antibiotic stewardship in intensive care. Lancet Infect Dis 2016;16:887–8. 27477972 10.1016/S1473-3099(16)30155-4

[21] de Jong E van Oers JA Beishuizen A Efficacy and safety of procalcitonin guidance in reducing the duration of antibiotic treatment in critically ill patients: a randomised, controlled, open-label trial. Lancet Infect Dis 2016;16:819–27. 26947523 10.1016/S1473-3099(16)00053-0

[22] Pupelis G Drozdova N Mukans M Serum procalcitonin is a sensitive marker for septic shock and mortality in secondary peritonitis. Anaesthesiol Intensive Ther 2014;46:262–73. 25293477 10.5603/AIT.2014.0043

[23] Eckmann C Sanchez-Garcia M Monitoring treatment response in abdominal sepsis with procalcitonin—if only!. Crit Care 2013;17:1017. 24326175 10.1186/cc13154 PMC4056891

[24] Sarbinowski R Arvidsson S Tylman M Plasma concentration of procalcitonin and systemic inflammatory response syndrome after colorectal surgery. Acta Anaesthesiol Scand 2005;49:191–6. 15715620 10.1111/j.1399-6576.2004.00565.x

[25] Lindberg M Hole A Johnsen H Reference intervals for procalcitonin and C-reactive protein after major abdominal surgery. Scand J Clin Lab Invest 2002;62:189–94. 12088337 10.1080/003655102317475443

[26] Jekarl DW Lee SY Lee J Procalcitonin as a diagnostic marker and IL-6 as a prognostic marker for sepsis. Diagn Microbiol Infect Dis 2013;75:342–7. 23391607 10.1016/j.diagmicrobio.2012.12.011

[27] Meisner M Tschaikowsky K Hutzler A Postoperative plasma concentrations of procalcitonin after different types of surgery. Intensive Care Med 1998;24:680–4. 9722037 10.1007/s001340050644

[28] Mokart D Merlin M Sannini A Procalcitonin, interleukin 6 and systemic inflammatory response syndrome (SIRS): early markers of postoperative sepsis after major surgery. Br J Anaesth 2005;94:767–73. 15849208 10.1093/bja/aei143

[29] Oberhofer D Juras J Pavicić AM Comparison of C-reactive protein and procalcitonin as predictors of postoperative infectious complications after elective colorectal surgery. Croat Med J 2012;53:612–9. 23275327 10.3325/cmj.2012.53.612 PMC3541587

[30] Garcia-Granero A Frasson M Flor-Lorente B Procalcitonin and C-reactive protein as early predictors of anastomotic leak in colorectal surgery: a prospective observational study. Dis Colon Rectum 2013;56:475–83. 23478615 10.1097/DCR.0b013e31826ce825

